# Case report: A case report and literature review on spontaneous bacterial peritonitis induced by intestinal barrier damage in a colorectal cancer patient with malnutrition

**DOI:** 10.3389/fonc.2025.1444149

**Published:** 2025-02-17

**Authors:** Peng Xu, Sanbao Shi, Zhiyu Yu, Da Li, Cheng Zhang

**Affiliations:** Department of General Surgery, General Hospital of Northern Theater Command, Shenyang, Liaoning, China

**Keywords:** spontaneous bacterial peritonitis, intestinal barrier, malnutrition, colorectal cancer, surgery

## Abstract

**Background:**

Spontaneous bacterial peritonitis (SBP) is an infectious condition characterizing the presence of bacterial infection in the peritoneal fluid with no apparent source of infection within the abdomen. It is extremely rare for patients with malnutrition after colorectal cancer (CRC) surgery to develop SBP. This is the first ever case reported case of SBP resulting from intestinal barrier compromise in a patient with colorectal cancer with malnutrition.

**Case summary:**

A 72-year-old woman with malnutrition was diagnosed with CRC, and following brief nutritional support, she underwent the laparoscopic-assisted radical right hemicolectomy. The patient was then diagnosed with peritonitis after the operation. An emergency laparotomy was performed, and the patient was finally diagnosed with SBP. The patient ultimately recovered following a series of appropriate postoperative supportive treatments.

**Conclusion:**

This case highlights the poor outcomes of short preoperative nutritional therapy in CRC patients with malnutrition. Further studies should investigate the role of the intestinal barrier function in the recovery of patients with CRC after surgery.

## 1 Introduction

Severe malnutrition results in intestinal villus blunting and compromises intestinal barrier function, which facilitates microbial translation, inducing systemic inflammation, potentially leading to sepsis (1). Moreover, chronic malnutrition-driven dysbiosis is characterized by an increased proportion of gram-negative bacteria, resulting in endotoxemia and the subsequent leakage of lipopolysaccharide (LPS) (2). Spontaneous bacterial peritonitis (SBP) arises from ascitic fluid infection without an evident intra-abdominal source, such as the gastrointestinal tract or other areas within the abdomen (3). Impaired intestinal barrier function and bacterial translocation (BT) are major clinical features in the pathogenesis of SBP (4). Therefore, malnutrition is known to promote the translocation of intestinal bacteria, and can potentially induce SBP. Here, we report a case of primary peritonitis in a patient with colorectal cancer (CRC) and malnutrition, resulting from intestinal barrier damage, and review literature to assess the interplay among peritonitis, malnutrition, and intestinal barrier damage.

## 2 Case presentation

**Figure 1:**
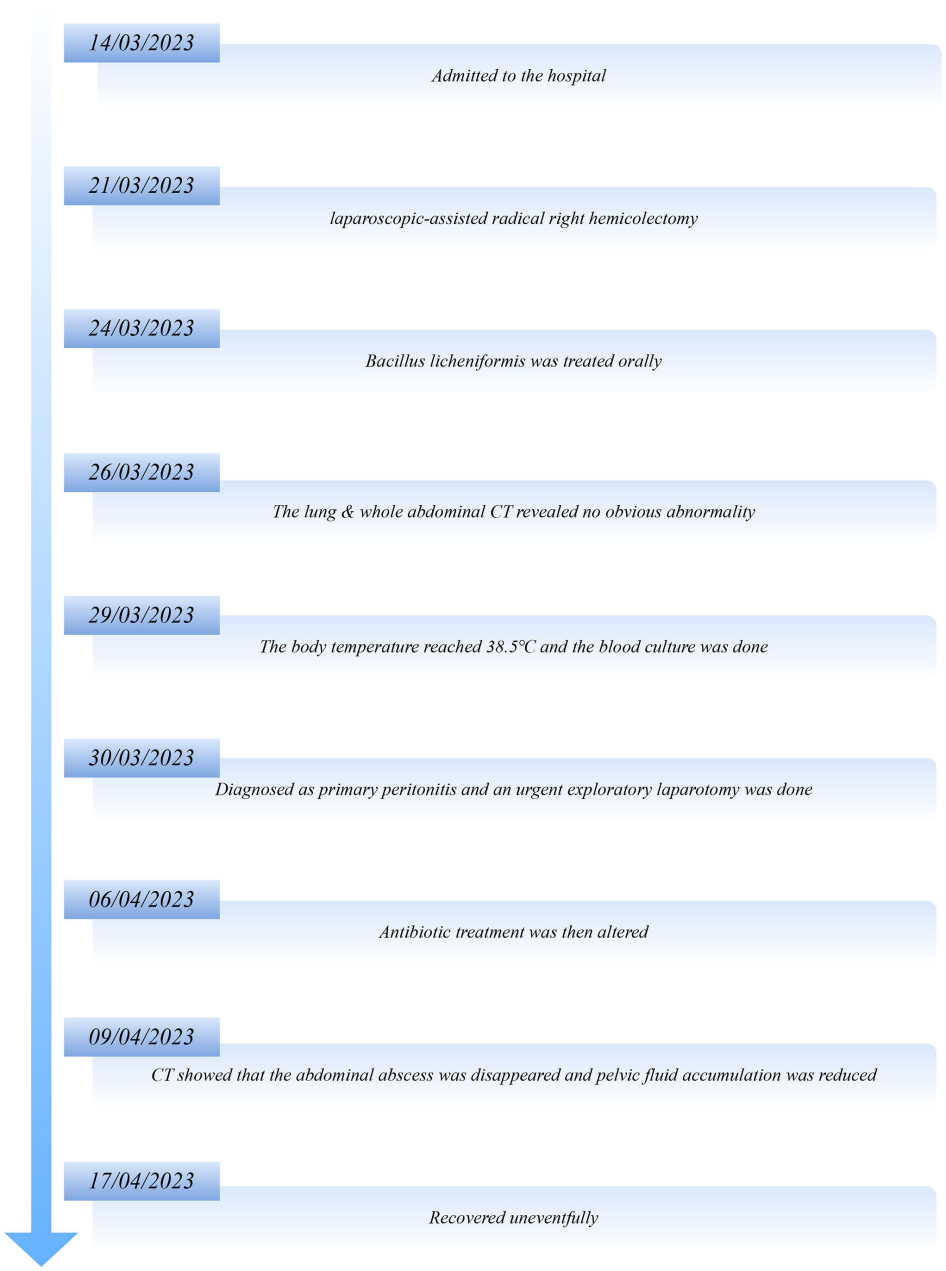
The timeline of the patient’s disease.

The timeline of the patient’s disease is shown in **Figure 1**.

### 2.1 Support therapy before surgery treatment

**Figure 2:**
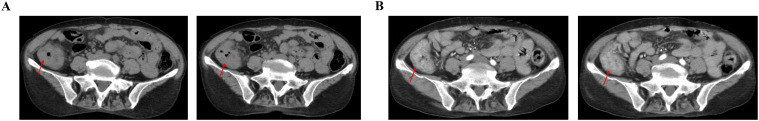
On 15 March 2023, CT showed the thickening of ascending colon duct wall with multiple surrounding lymph nodes. **(A)** Plain scan of abdominal CT image. **(B)** Enhancement scan of abdominal CT image.

A 72-year-old female diagnosed with ‘bacterial pneumonia’ was admitted to the local hospital on 12 February 2023. The patients had an 8-year neurosis history and had been given olanzapine and paroxetine hydrochloride oral treatment. An admission examination revealed gastrointestinal bleeding. Colonoscopy revealed an irregular mucosal elevation around the circumference about 80cm from the anus (ascending colon near the ileocecal valve), characterized by an ulcerated and uneven surface, covered with exudates, and exhibiting bleeding and erosion. The tissues appeared fragile and bled easily upon contact. Pathologic diagnosis was ‘high-grade intraepithelial neoplasia (colon), intraepithelial carcinoma (intramucosal carcinoma)’. The patient subsequently presented to our hospital for additional surgical intervention on 14 March 2023. The body mass index (BMI) of the patient was 17.58 kg/m^2^ (height: 1.6 m, weight: 45 kg). The patient lost 30 kg weight in the last 2 years. According to the ESPEN recommendations, the patient was diagnosed with malnutrition (5). Abdomen computed tomography (CT, routine & enhanced scan) showed the thickening of the ascending colon duct wall and the presence of multiple surrounding lymph nodes (**Figures 2A, B**). The hemoglobin (HGB) and hematocrit (HCT) of the patient were 59 g/L and 0.197 L/L. Prealbumin (PA) was 215.8 mg/L and albumin (ALB) was 32.5 g/L. After a week of treatment with blood transfusions and nutritional support therapy, there was a significant improvement in patients’ anemia and nutritional status (HGB:105 g/L, HCT:0.327 L/L, PA:230.2 mg/L, ALB:33.6 g/L) (**Supplementary Figures S1**, **S2**). After the preoperative preparation, the patient proceeded to undergo laparoscopic-assisted radical right hemicolectomy under general anesthesia on 21 March 2023. The postoperative pathological diagnosis revealed no involvement of lymph nodes. The grade for the CRC was T4aN0M0 IIB. The body temperature and levels of inflammatory markers during hospitalization are listed in **Supplementary Table S1**, **Supplementary Figure S3**.

### 2.2 Spontaneous bacterial peritonitis after surgery

**Figure 3:**
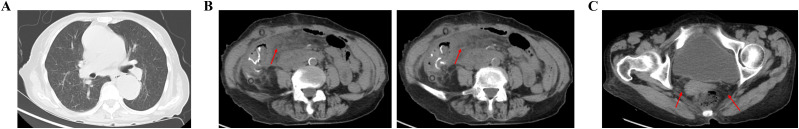
On 26 March 2023, CT showed no obvious abnormality in the lungs, abdominal and pelvic cavity. **(A)** Chest routine scan of CT image. **(B)** Abdomen routine scan of CT image. **(C)** Pelvic routine scan of CT image.

**Figure 4:**
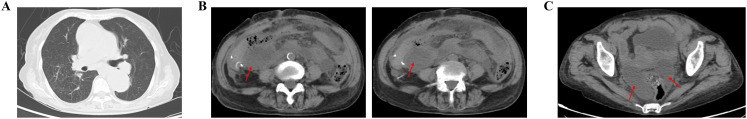
On 30 March 2023, CT showed the abdominal abscess and pelvic effusion were formatted. **(A)** Chest routine scan of CT image. **(B)** Abdomen routine scan of CT image. **(C)** Pelvic routine scan of CT image.

On the 3^rd^ (24 March 2023) postoperative day, the patient began to flatus for four times and had a bowel movement, and then was fed with full fluids. Concurrent with other treatments, *Bacillus licheniformis* was administered orally to modulate the gut microbiota. The patient experienced intermittent fever, reaching a maximum temperature of 38°C in the postoperative period. Hence, a comprehensive CT scan of the lung and the whole abdomen was performed on 26 March 2023, which revealed no obvious abnormality in the abdominal cavity (**Figures 3A, B**). Moreover, pelvic cavity assessment revealed no fluid accumulation (**Figure 3C**). The abdominal drainage tube was subsequently removed. On the 6^th^ postoperative night (27 March 2023), the patient experienced diarrhea six times (each time expelling approximately 100 ml) and subsequently developed abnormal liver and kidney functions. The patient was administered rehydration and other symptomatic treatments. Over the subsequent 2 days, the patient experienced intermittent low fevers, with temperatures not exceeding 38°C. On the 8^th^ day (29 March 2023), the patient got a fever, with a body temperature of 38.5°C. A complete blood count (CBC) was conducted (**Supplementary Table S1**), and levels of inflammatory markers were measured as follows: Procalcitonin (PCT) 10.87 ng/mL, Interleukin-6 (IL-6) 337.73 pg/mL, and high-sensitivity C-reactive protein (hCRP) 144.8 mg/L. Blood cultures were additionally performed. A 10 mL blood sample was drawn from the patient, and *B. licheniformis* was identified within 23 h. Imipenem was subsequently administered to treat the infection. CT on the 9th postoperative day (30 March 2023) revealed the abdominal abscess and pelvic effusion (**Figures 4B, C**). However, there were no significant signs of inflammation in the lungs (**Figure 4A**). Physical examination demonstrated generalized abdominal tenderness and the presence of rebound pain in the patient. The heart rate of patient was 98 beat per minute (bpm), the blood pressure was 152/67 mmHg, and the oxygen saturation was 98%. The patient had symptoms of infection and was then diagnosed with peritonitis. Considering the possibility of an anastomotic fistula, an urgent exploratory laparotomy was performed. Intraoperatively, a significant quantity of purulent exudate and fibrinous deposits was found coating the small intestine in the abdominal cavity, and approximately 50 mL of purulent fluid was aspirated from the pelvis and transferred to a blood culture bottle for enrichment. The polymorphonuclear (PMN) count in the ascitic fluid exceeded 250 cells per mm^3^, although the fluid culture ultimately yielded no growth. Moreover, exploration of the anastomosis revealed no abnormalities. As a result, abdominal lavage and drainage were conducted. Postoperatively, the patient was transferred to the emergency intensive care unit on 30 March 2023. Finally, the patient was diagnosed as an SBP based on established diagnostic criteria (6).

### 2.3 Adjuvant treatment after second surgery

After 2 days (1 April 2023), the patient was transferred back to the general surgery department. Within the initial 4 days, the patient exhibited intermittent fever, with the maximum temperature reaching 38°C. The patient was able to tolerate a small amount of water without experiencing abdominal pain and bloating, and there was no defecation, and three abdominal drains without fluid drainage. The bacterial/fungal culture of blood was performed on 4 April 2023 and the results were negative. The fecal coccus-to-bacillus ratio on 5 April 2023 was 1:1. On 6 April 2023, the patient passed approximately 200 ml of dark red, bloody stool. Related indicators of coagulation were: prothrombin time (PT) 26.9s; international standardized ratio (INR) 2.46; prothrombin activity (PTA) 29%; activated partial thromboplastin time (APTT) 79.8 s; D-dimer 8.48mg/L FEU. Antibiotic treatment was then altered to omadacycline, caspofungin acetate, and tinidazole after consulting the clinical pharmacist. Moreover, the vancomycin was administered orally. Ten days after the second surgery, the CT plain scan was checked on 9 April 2023. CT showed that the abdominal abscess had resolved, and the pelvic fluid accumulation had reduced (**Supplementary Figures S4A, B**). Finally, all three drainage tubes were progressively removed over the subsequent 8 days, leading to the recovery of the patient (17 April 2023).

## 3 Discussion

CRC is still one of the most prevalent cancers and a major cause of the global rise in cancer-related deaths (7). With the advent of new surgical instruments and methods, as well as the exponential growth of robotic surgery, the surgical treatment of CRC has changed over the past 10 years (8). Postoperative recovery efforts, primarily directed at interventions and intraoperative factors, contribute significantly to enhancing patient outcomes after surgery (9). The key characteristics associated with CRC include low food intake, impaired nutrient absorption, and heightened systemic inflammation are key characteristics associated with CRC, leading to a state of increased catabolism and decreased anabolism, which defines malnutrition (10). Previous studies on gastric cancer have shown shorter survival among patients with malnutrition before surgery (11). Malnutrition rates among patients with CRC might range from 20% to 37%, depending on the method used for nutritional status assessment (12). Malnutrition is an independent risk factor of CRC, which is associated with poorer clinical outcomes for patients (13). In addition, malnutrition and low muscle mass are independently associated with poor outcomes in affected patients (13). Xinying Wang et al. demonstrated a correlation between the PG-SGA score and the prediction of post-operative infection in patients with CRC (14). Patients with CRC commonly face nutritional risk or malnutrition, exacerbated by the physiological stress of surgery (15). Malnutrition significantly affects their tolerance of treatment, and contributes to postoperative complications, including anastomotic leakage (AL), which in turn impacts oncological outcomes (16). In the current case, the occurrence of SBP following colorectal surgery in a malnourished patient was relatively uncommon, moreover, no common postoperative complications (such as AL) were observed.

### 3.1 Reasons underlying the development of spontaneous bacterial peritonitis after operation

Chloé Magnan et al. observed a significant reduction in both the abundance as well as the diversity of the gut microbiota following digestive surgery, with a concomitant significant increase in the abundance of the *genus Enterococcus* (17). Our patient underwent laparoscopic-assisted radical right hemicolectomy. Previous studies showed that the incidence of surgical site infection (SSI) is as low as 2.1% when compared to open laparotomy surgery (18). Probiotics therapy post-surgery has been demonstrates significantly reduced levels of pro-inflammatory cytokines in blood serum and alleviates side effects following CRC surgery (19). Probiotic and symbiotic therapies have been proven to enhance recovery of patients after CRC surgery through the reduction in intestinal permeability and lowering the incidence of infection (20). Although our patient took *B. licheniformis* orally after surgery, the occurrence of SBP could not be prevented. The pathogenesis of SBP involves impaired intestinal barrier function, potentially exacerbated by BT and malnutrition. BT characterizes the translocation of intestinal microbes and their products from the intestinal lumen to sterile sites, including circulating blood, mesenteric lymph nodes, abdominal cavity, and internal organs (21). Impaired intestinal integrity is associated with BT-mediated systemic inflammation and sepsis consequently leading to clinical deterioration and possible death (1). The blood culture with *B. licheniformis* in blood also demonstrated the impairment of the intestinal barrier and BT.

Finally, an impaired intestinal barrier and BT can result in SBP in patients. A earlier study showed markedly increased intestinal aerobic bacterial count due to BT and SBP (22). In our patient, overgrowth of intestinal bacteria was associated with an increased risk of the BT and SBP; therefore, oral administration of vancomycin was effective following the second operation. Apart from malnutrition, severe anemia is negatively correlated with the intestinal barrier and is known to trigger BT, co-occurs with CRC and was identified in our patient (23). Moreover, BT is reported to be more frequent in patients receiving total parenteral nutrition (TPN) or postoperatively (24), mainly due to the compromised integrity of the intestinal barrier during the TPN therapy (24). Malnutrition and anemia jointly contribute to the intestinal barrier impairment, which in turn leads to the occurrence of SBP.

### 3.2 Clinical value of malnutrition in intestinal barrier

The intestinal mucosa is located in the innermost layer of the digestive tract. It consists of the epithelium, the lamina propria, and the muscularis mucosae (25), and acts as the intestinal barrier to regulate intestinal homeostasis (26). Dietary components interact with the intestinal lumen and have been found to regulate intestinal permeability (27). Malnutrition refers to a deficiency in energy, protein, and other nutrients that results in observable negative effects on tissue and body form (body shape, size, and composition), function, and clinical outcome (28). Jacobi et al. demonstrated that malnutrition is linked to a significant decrease in villus height and transepithelial electrical resistance (29). Consistently, caloric restriction and undernutrition also result in villus atrophy (30). Moderate acute malnutrition (MAM) has been demonstrated to elevate systemic inflammation of mice and is associated with increased bioactivity of BT and bacterial LPS in the intestine, ultimately leading to inflammatory responses (2). Another study also showed that the malnutrition-enteric diet is associated with an increased Lactulose: Mannitol ratio (LM) and a reduction in the villous area of the intestinal epithelial barrier (31). Maghraby et al. demonstrated that low-protein diet induced intestinal villus atrophy and compromised the intestinal barrier (32). Moreover, the levels of CLD-3 and CLD-4 proteins also reduce, while occludin level is enhanced, and mitochondrial function is impaired in response to LPD in intestinal epithelial cells (IECs) (1). Disruption of the epithelial barrier allows bacteria and bacterial components to enter the epithelium, initiating immunological responses (33). The integrity of the intestinal barrier is crucial for mitigating postoperative complications. Indeed, patients with malnutrition could benefit from appropriate nutritional support to improve the intestinal barrier function prior to CRC surgery.

## 4 Conclusion

This case demonstrated SBP resulting from intestinal barrier compromise in a patient with malnutrition following surgery for CRC. Further research should focus on the role of intestinal barrier function in the postoperative recovery of patients with CRC. Additionally, the integrity of the intestinal barrier is crucial for patients undergoing surgery for CRC.

## Data Availability

The raw data supporting the conclusions of this article will be made available by the authors, without undue reservation.

## Supplementary material

Supplementary Figure 1 Levels of serum RBC, HGB, and HCT before operation. **(A)** Levels of serum RBC. **(B)** Levels of serum HGB. **(C)** Levels of serum HCT.

Supplementary Figure 2 Levels of serum PA, ALB, and TP before operation. **(A)** Levels of serum PA. **(B)** Levels of serum ALB. **(C)** Levels of serum TP.

Supplementary Figure 3 Levels of inflammatory markers and the body temperature. **(A)** Levels of WBC. **(B)** Levels of NEU. **(C)** Levels of PCT. **(D)** Body temperature of the patient.

Supplementary Figure 4 On 9 April 2023, CT showed that the abdominal abscess had disappeared, and pelvic fluid accumulation was reduced. **(A)** Abdomen routine scan of CT image. **(B)** Pelvic routine scan of CT image.

Supplementary Table 1 The levels of inflammatory markers in serum during the case.
