# Supplementary figures and images for: Case report: A case report and literature review on spontaneous bacterial peritonitis induced by intestinal barrier damage in a colorectal cancer patient with malnutrition
Source: Front Oncol. 2025 Feb 17;15:1444149. doi: 10.3389/fonc.2025.1444149 (PMC11872932; doi:10.3389/fonc.2025.1444149)

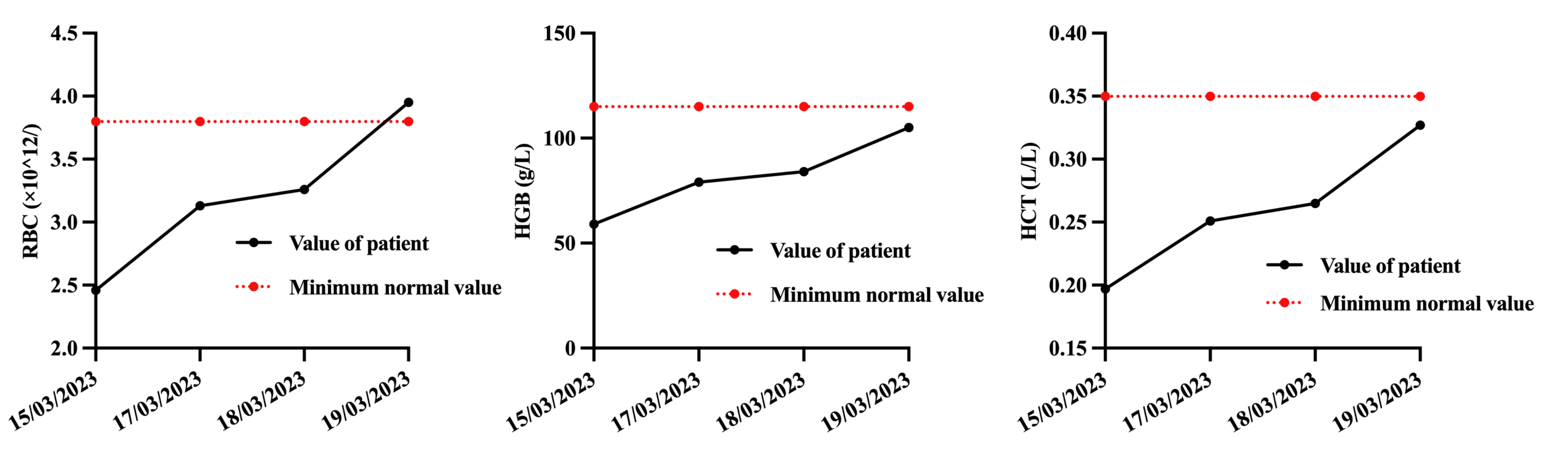

Supplement: Supplementary Figure 1 — Levels of serum RBC, HGB, and HCT before operation. (A) Levels of serum RBC. (B) Levels of serum HGB. (C) Levels of serum HCT. [file Image1.tif]

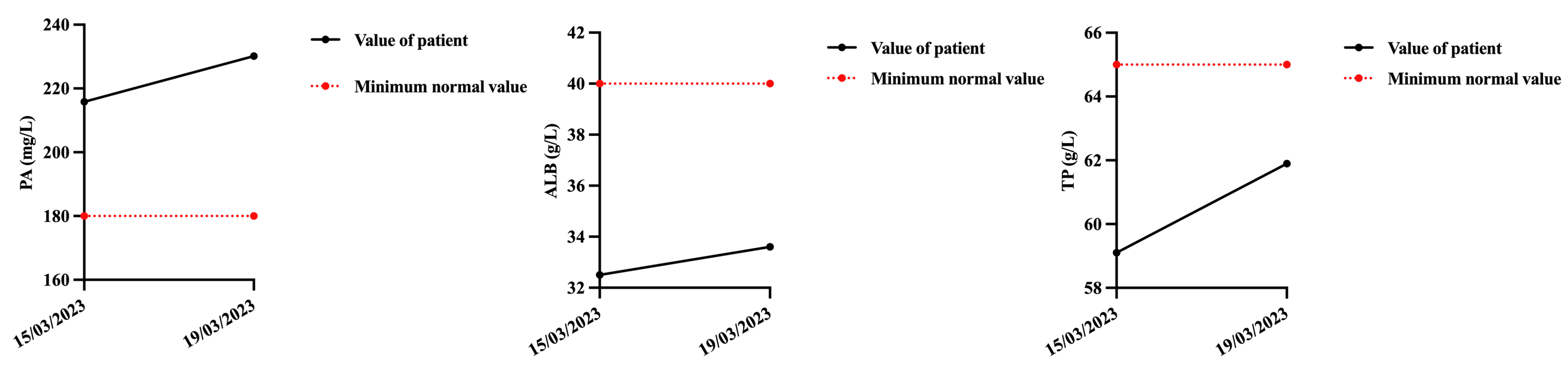

Supplement: Supplementary Figure 2 — Levels of serum PA, ALB, and TP before operation. (A) Levels of serum PA. (B) Levels of serum ALB. (C) Levels of serum TP. [file Image2.tif]

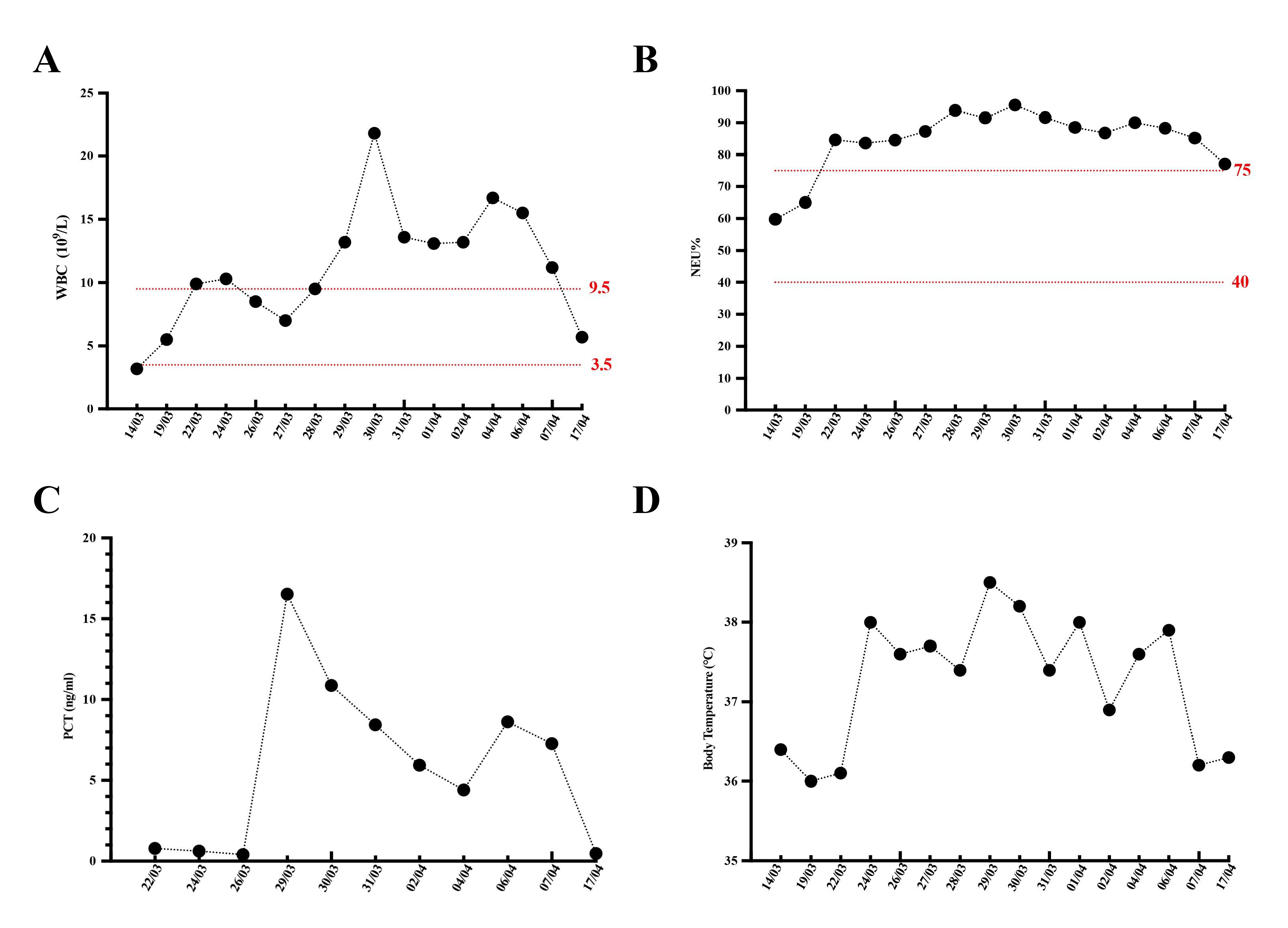

Supplement: Supplementary Figure 3 — Levels of inflammatory markers and the body temperature. (A) Levels of WBC. (B) Levels of NEU. (C) Levels of PCT. (D) Body temperature of the patient. [file Image3.tif]

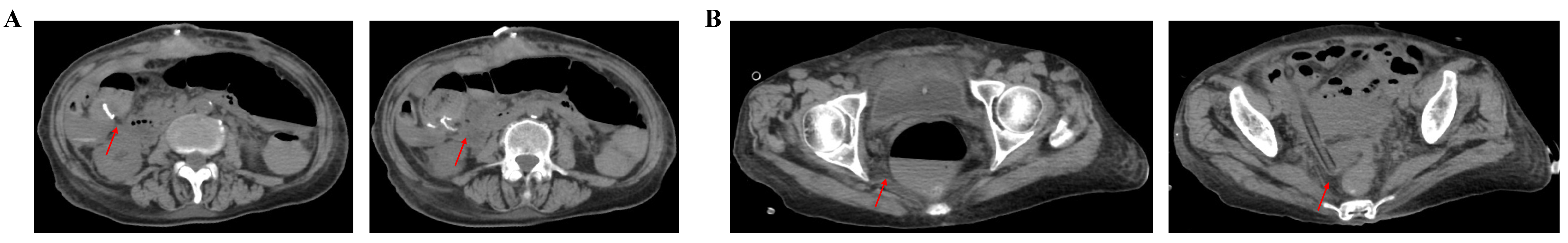

Supplement: Supplementary Figure 4 — On 9 April 2023, CT showed that the abdominal abscess had disappeared, and pelvic fluid accumulation was reduced. (A) Abdomen routine scan of CT image. (B) Pelvic routine scan of CT image. [file Image4.tif]
