# Supplementary material for: Case report: A case report and literature review on spontaneous bacterial peritonitis induced by intestinal barrier damage in a colorectal cancer patient with malnutrition
Source: Front Oncol. 2025 Feb 17;15:1444149. doi: 10.3389/fonc.2025.1444149 (PMC11872932; doi:10.3389/fonc.2025.1444149)
Supplement: Supplementary Table 1 — The levels of inflammatory markers in serum during the case. [file Table1.docx]

**Table 1. The levels of inflammatory markers in serum during the case**

| **Data** | **Events** | **WBC(×10^9^/L)**  **(3.5-9.5) ×10^9^/L** | **NEU%**  **(40.0-75.0) %** | **PCT**  **(0.00-0.05) ng/ml** | **LY%**  **(20.0-50.0) %** | **MO%**  **(3.0-10.0) %** | **EO%**  **(0.4-8.0) %** | **BA%**  **(0.0-1.0) %** | **NEU (×10^9^/L)**  **1.80-6.30 (×10^9^/L)** | **LY(×10^9^/L)**  **1.10-3.20 (×10^9^/L)** | **MO(×10^9^/L)**  **0.10-0.60 (×10^9^/L)** | **EO(×10^9^/L)**  **0.02-0.52 (×10^9^/L)** | **BA(×10^9^/L)**  **0.00-0.06 (×10^9^/L)** |
| --- | --- | --- | --- | --- | --- | --- | --- | --- | --- | --- | --- | --- | --- |
| 14/03/2023 | Admitted to hospital | 3.2 | 59.8 | NA | 25.2 | 10.3 | 4.3 | 0.4 | 1.91 | 0.81 | 0.33 | 0.14 | 0.01 |
| 19/03/2023 | Preoperative | 5.5 | 65 | NA | 23.1 | 9 | 2.7 | 0.2 | 3.58 | 1.27 | 0.5 | 0.15 | 0.01 |
| 22/03/2023 | 1st day after operation | 9.9 | 84.6 | 0.79 | 9.2 | 5.8 | 0.3 | 0.1 | 8.38 | 0.91 | 0.57 | 0.03 | 0.01 |
| 24/03/2023 | 3rd day after operation | 10.3 | 83.6 | 0.63 | 10 | 5.7 | 0.6 | 0.1 | 8.61 | 1.03 | 0.59 | 0.06 | 0.01 |
| 26/03/2023 | 5th day after operation | 8.5 | 84.5 | 0.41 | 4.8 | 8.8 | 1.8 | 0.1 | 7.18 | 0.41 | 0.75 | 0.15 | 0.01 |
| 27/03/2023 | 6th day after operation | 7 | 87.3 | NA | 5.9 | 6.7 | 0.1 | 0 | 6.11 | 0.41 | 0.47 | 0.01 | 0 |
| 28/03/2023 | 7th day after operation | 9.5 | 93.9 | NA | 2.6 | 3.4 | 0.1 | 0 | 8.92 | 0.25 | 0.32 | 0.01 | 0 |
| 29/03/2023 | 8th day after operation | 13.2 | 91.5 | 16.53 | 3.6 | 4.8 | 0.1 | 0 | 12.08 | 0.48 | 0.63 | 0.01 | 0 |
| 30/03/2023 | 9th day after operation | 13.3 | 87.8 | 11.79 | 5.3 | 6.8 | 0.1 | 0 | 11.68 | 0.7 | 0.9 | 0.01 | 0 |
| 30/03/2023 | Day of second operation | 21.8 | 95.5 | 10.87 | 1.6 | 2.9 | 0 | 0 | 20.82 | 0.35 | 0.63 | 0 | 0 |
| 31/03/2023 | 1st after second operation | 13.6 | 91.6 | 8.44 | 3.6 | 4.8 | 0 | 0 | 12.46 | 0.49 | 0.65 | 0 | 0 |
| 01/04/2023 | 2nd after second operation | 13.1 | 88.5 | NA | 5.4 | 6.1 | 0 | 0 | 11.59 | 0.71 | 0.8 | 0 | 0 |
| 02/04/2023 | 3rd after second operation | 13.2 | 86.8 | 5.94 | 7 | 6.1 | 0.1 | 0 | 11.46 | 0.92 | 0.81 | 0.01 | 0 |
| 04/04/2023 | 5th after second operation | 16.7 | 90 | 4.41 | 5 | 4.9 | 0.1 | 0 | 15.03 | 0.84 | 0.82 | 0.02 | 0 |
| 06/04/2023 | 7th after second operation | 15.5 | 88.3 | 8.63 | 6.5 | 5.2 | 0 | 0 | 13.69 | 1.01 | 0.81 | 0 | 0 |
| 07/04/2023 | 8th after second operation | 11.2 | 85.2 | 7.28 | 8.2 | 6.5 | 0 | 0.1 | 9.54 | 0.92 | 0.73 | 0 | 0.01 |
| 08/04/2023 | 9th after second operation | 13.6 | 87.1 | NA | 5.7 | 7.1 | 0 | 0.1 | 11.85 | 0.78 | 0.97 | 0 | 0.01 |
| 09/04/2023 | 10th after second operation | 10.8 | 83.8 | 4.82 | 8.7 | 7.4 | 0 | 0.1 | 9.05 | 0.94 | 0.8 | 0 | 0.01 |
| 10/04/2023 | 11th after second operation | 10.7 | 84.3 | 5.22 | 7.5 | 8 | 0 | 0.2 | 9.02 | 0.8 | 0.86 | 0 | 0.02 |
| 17/04/2023 | Recovered uneventfully | 5.7 | 77.1 | 0.48 | 11.3 | 10.6 | 0.8 | 0.2 | 4.39 | 0.64 | 0.6 | 0.05 | 0.01 |

WBC:White Blood Cell; NEU:Neutrophils; PCT:Procalcitonin; LY:Lymphocyte; MO:Monocyte;EO:**Eosinophil;** BA:**Basophil**
